# Combating cholera by building predictive capabilities for pathogenic Vibrio cholerae in Yemen

**DOI:** 10.1038/s41598-022-22946-y

**Published:** 2023-02-08

**Authors:** Moiz Usmani, Kyle D. Brumfield, Bailey M. Magers, Juan Chaves-Gonzalez, Helen Ticehurst, Rosa Barciela, Fergus McBean, Rita R. Colwell, Antarpreet Jutla

**Affiliations:** 1 GeoHealth and Hydrology Laboratory, Department of Environmental Engineering Sciences, University of Florida, Gainesville, FL USA; 2 Maryland Pathogen Research Institute, University of Maryland, College Park, MD 20742 USA; 3 Institute for Advanced Computer Studies, University of Maryland, University of Maryland, College Park, MD 20742 USA; 4 United Nations Office for the Coordination of Humanitarian Affairs, New York, NY USA; 5 Meteorological Office, Exeter, UK; 6 Foreign, Commonwealth and Development Office, London, UK

**Keywords:** Climate sciences, Environmental sciences, Diseases, Risk factors

## Abstract

Cholera remains a global public health threat in regions where social vulnerabilities intersect with climate and weather processes that impact infectious *Vibrio cholerae*. While access to safe drinking water and sanitation facilities limit cholera outbreaks, sheer cost of building such infrastructure limits the ability to safeguard the population. Here, using Yemen as an example where cholera outbreak was reported in 2016, we show how predictive abilities for forecasting risk, employing sociodemographical, microbiological, and climate information of cholera, can aid in combating disease outbreak. An epidemiological analysis using Bradford Hill Criteria was employed in near-real-time to understand a predictive model’s outputs and cholera cases in Yemen. We note that the model predicted cholera risk at least four weeks in advance for all governorates of Yemen with overall 72% accuracy (varies with the year). We argue the development of anticipatory decision-making frameworks for climate modulated diseases to design intervention activities and limit exposure of pathogens preemptively.

## Introduction

Cholera, is a signature dehydrating diarrheal disease transmitted notably via untreated drinking water carrying the causative agent, *Vibrio cholerae*^1^. The disease has plagued humans for thousands of years, with reports of cholera-like symptoms documented in Sanskrit medical texts ~ 500–400 B.C.^2^. While the sporadic seasonal outbreak has been reported throughout history^1^, the first cholera pandemic was reported between 1817 and 1823. Globally, cholera has continued to spread, and the ongoing pandemic, the seventh, which began in 1961, is caused by the El Tor biotype of *V. cholerae* O1. While *V. cholerae *non-O1 serogroups are not known to cause epidemics of diarrhea, sporadic cases and small outbreaks of diarrhea and extraintestinal infections have been reported. Early environmental studies of cholera were unsuccessful in detecting reservoirs of *V. cholerae*, such as domestic animals or human carriers^3^, until the late 1960s, when the bacterium was detected in environmental water samples collected in cholera-free regions^4,5^. Those *V. cholerae* were subsequently shown to be associated with zooplankton^6–8^. Thereafter, the environmental source of *V*. *cholerae* was demonstrated extensively in studies carried out from 1970 to 2000 in countries around the world^1,9–12^. However, between outbreaks and unfavorable environmental conditions, the bacterium persists in environmental reservoirs, commonly in a viable, but non-culturable state^13^.. Warmer sea surface and coastal water temperatures have been identified as drivers of *V. cholerae* prevalence in the environment and are associated with increased numbers of cholera cases^9,14,15^. Several environmental and climatic variables have been linked to the proliferation of *V. cholerae* and increased incidence of cholera, such as precipitation^16^, flooding^17^, sea surface temperature and height^9^, river level and freshwater discharge^14^, coastal salinity^18^, dissolved organic material^19^, chlorophyll^20^, and components of phytoplankton and zooplankton populations^20^. In addition, recent epidemiological surveillance suggests a link with estuarine ecosystems, namely river and coastal regions^21^.

**Figure 1:**
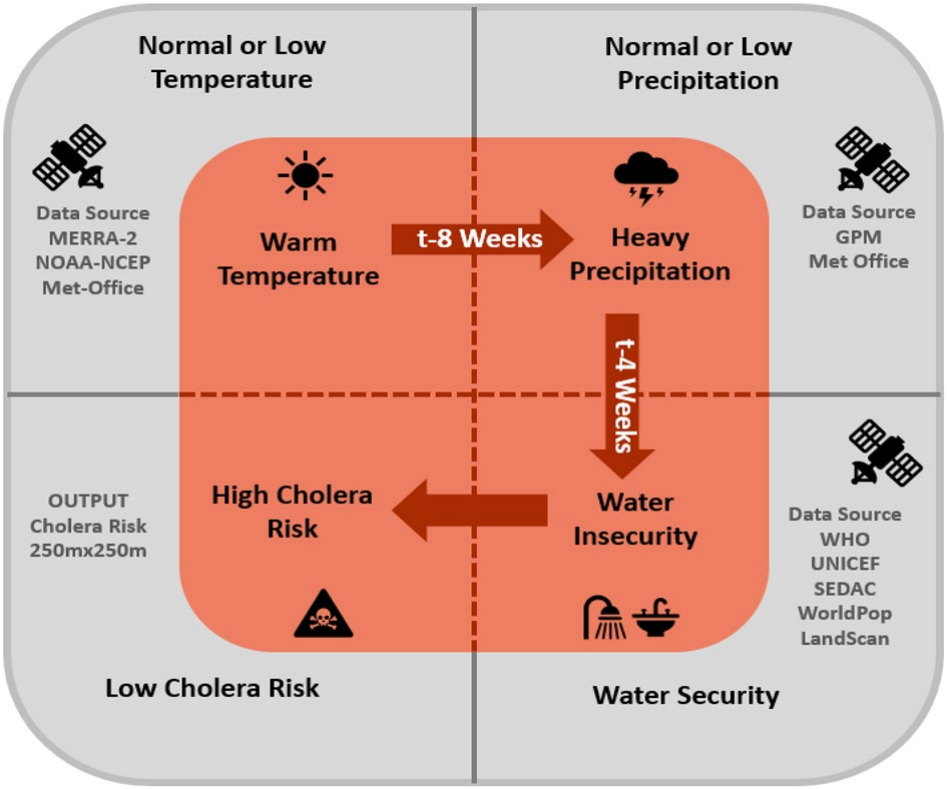
Cholera trigger mechanism. Adapted from “Environmental Factors Influencing Epidemic Cholera”, by Jutla et al., 2013, The American journal of tropical medicine and hygiene 89 (3), 597–607.

Based on analysis of cholera records maintained in India from 1823 to 1875, cholera has been defined as occurring in two dominant forms: (1) epidemic, characterized by the sudden and sporadic occurrence of a large number of cases; and (2) endemic, where cholera cases occur at a baseline level throughout the year with distinct seasonal peaks^22,23^. Epidemic cholera is hypothesized to be related to elevated air temperatures followed by above-average precipitation in concatenation with insufficient and/or damaged water, sanitation, and hygiene (WASH) infrastructure, placing the human population at high risk of interaction with cholera bacteria and a subsequent disease outbreak^24^ (Fig. 1). Endemic cholera is associated with the constant occurrence of cholera cases, primarily in regions where coastal or terrestrial water systems create favorable conditions for the growth and proliferation of *V. cholerae.* Under certain environmental conditions, a sustained epidemic mode of cholera can evolve into the endemic form in regions with enhanced and continuing exposure to, and transmission of, *V. cholerae*.

As a pandemic disease, cholera affects millions in vulnerable human populations^25^ and persists as a dominant water-borne disease in Latin America, sub-Saharan Africa, and Southern Asia^26,27^. Massive cholera outbreaks are associated with natural and anthropogenic disasters, notably when environmental conditions favor the growth of the bacterium^28^. The cholera outbreak in Haiti, which occurred during the months following Hurricane Matthew^22^, serves as a prime example. Since March 2015, Yemen, a coastal Middle Eastern country, has suffered violent surges of civil unrest^29^, and in October 2016, the country reported a few cholera cases. By the end of 2017, Yemen accounted for *ca*. 80% of cholera cases worldwide since 2015^30^. During the first six months of the outbreak, cholera in Yemen surpassed the number of reported cases in Haiti over a span of seven years (*ca*. 815,000 cases between 2010 and 2017), now considered historically the largest cholera epidemic^31^.

Cholera is unlikely to be eradicated since the disease-causing agent is autochthonous to aquatic environments and plays a role in those environments' carbon and nitrogen cycles^20^. Clearly, the ecology of *V. cholerae* must be understood in the context of its natural aquatic habitat and the changing climate, hence a driver of cholera as a potential re-emerging infectious disease.

Disease prediction can be achieved by recognizing that disease progression comprises two components, trigger and transmission, which result in an outbreak and, subsequently, public health emergency^23,32^. For cholera, "trigger" represents those mechanisms that stimulate the growth, multiplication, and distribution of *V. cholerae* bacteria in the environment. Water insecurity, namely lack of access to safe water and sanitation, enhances the bacterium's interaction with human populations. Per contra, "transmission" comprises pathways that allow the dissemination of *V. cholerae* and involves complex interaction routes between humans and contaminated water^22^.

In our previous research, protocols were developed to predict cholera in various regions throughout the world^9,16,33–37^. The hypothesis shown in Fig. 1 was validated using retrospective data from countries in Africa and Asia over a decadal timeframe, most recently in Ukraine^38^. However, reliable spatial and temporal datasets containing disease prevalence or incidence time series are a major challenge for infectious disease prediction. Without consistent time series datasets, our previous studies^24,39,40^ relied on spatial pattern recognition principles to identify hydroclimatic and environmental processes associated with cholera trigger across geographical regions of interest. Yemen provided a unique opportunity because spatial and temporal cholera prevalence datasets were available from the beginning of the cholera epidemic up to the present, allowing model validation. One of the significant gaps to control cholera is inherent to the absence of knowledge on when and where an outbreak is likely to occur. The World Health Organization (WHO) Global Task Force on Cholera Control (GTFCC) initiative aims to reduce cholera deaths by 90% and eliminate cholera in at least 20 countries by 2030^41^. While vaccines^42^ and other interventions, e.g., removal of zooplankton and particulates by filtration^43,44^ and access to WASH infrastructure^45^ are critical to fight cholera disease, a predictive capability and capacity will likely provide an additional toolset for combating cholera outbreaks globally^1^. This reflective study presents an overview of the integration of microbiology of cholera used to develop a predictive system that assists aid agencies, namely by reporting cholera risk. Therefore, the objective of this study was to determine the evolution of cholera in Yemen, with the specific aim to validate the cholera trigger using the well-established epidemiological Bradford Hill Criteria (BHC) for causation^46,47^. The ultimate goal is to provide assistance in enhancing epidemiological and medicine-based decision-making so that future cholera outbreaks can be prevented.

## Results and discussion

**Figure 2:**
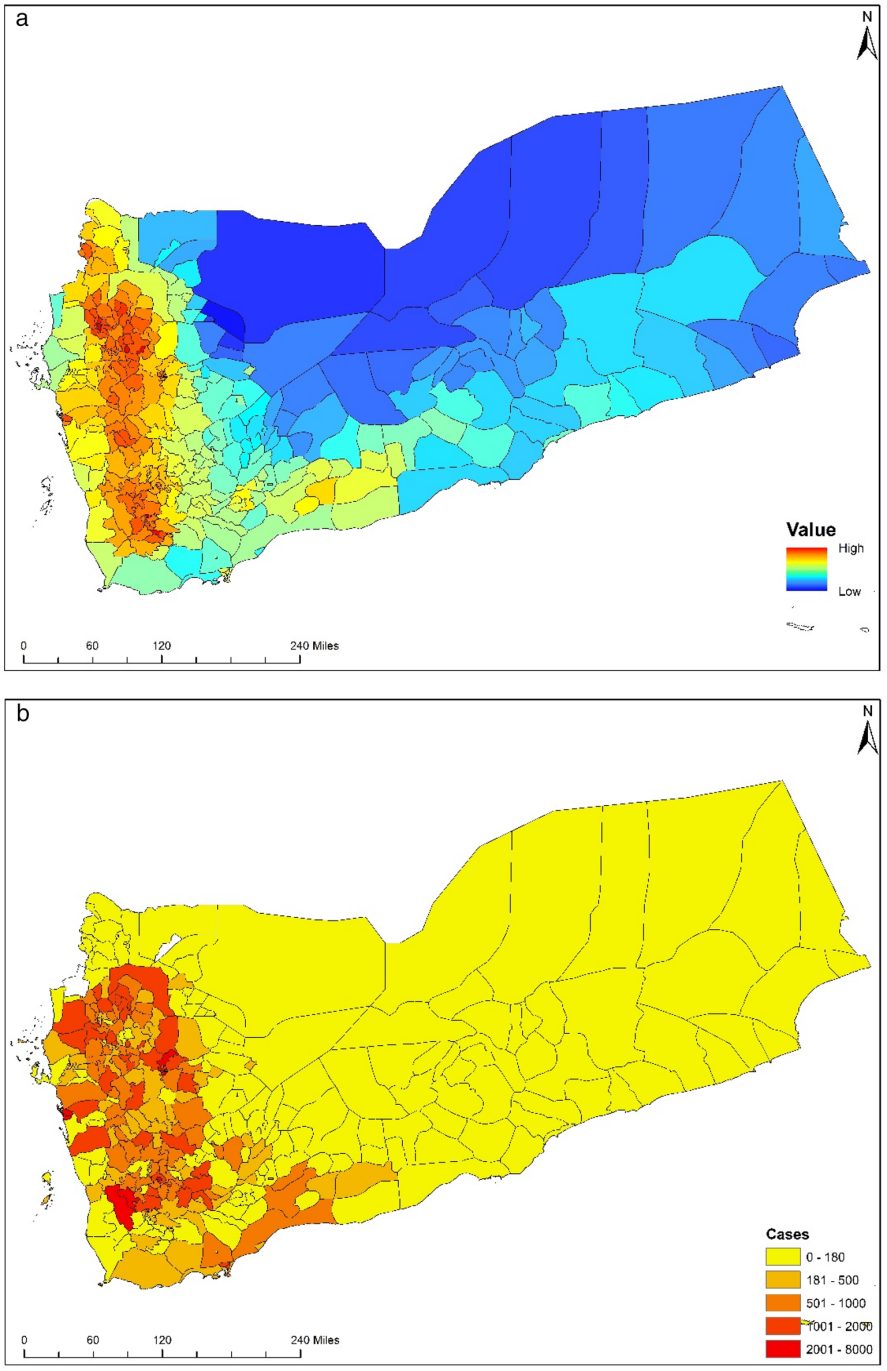
(**a**) Cholera risk map of Yemen for June, 2017 produced on May 30, 2017; (**b**) Actual cholera cases observed in June, 2017, maps are generated using ESRI’s ArcMap version 10.7 (URL provided in supplementary material).

The objective of this study is to evaluate the performance of a prediction system for cholera, employing near real-time data collected over the past three years by the Yemeni governorates and using Bradford Hill Criteria for the epidemiological association. The first indication of cholera in Yemen was noticed in October 2016. Subsequently, epidemiological incidence data became available in June 2017. The Cholera Risk Model (CRM) trigger module captured the increased risk of a massive cholera outbreak in Yemen (Fig. 2) with *ca*. 92% spatial match between locations where cholera cases were reported, and high risk was computed. The trigger component of the CRM had been validated previously, using historical data for Sudan, Bangladesh, Mozambique, Zimbabwe, Cameroon, and Haiti^22,24,39^. However, Yemen was unique since epidemiological conditions allowed for assessment of model performance in near real-time. BHC^46,48^, a set of ten parameters intended to provide epidemiological evidence for causal relationships between a public health outcome and factors influencing said outcome, was used as defining criteria for CRM performance. The following sub-sections detail each parameter of the BHC for model performance.

### Strength

Strength is a parameter of BHC that provides epidemiological evidence for the associational relationship between disease prevalence and factors influencing disease outbreaks. Furthermore, the climate has been established as a driver of cholera^24,49^, and temperature and rainfall facilitate the growth and proliferation of the bacterium and enhance its metabolic activity. Cholera infections have a hallmark seasonal distribution, with most cases occurring during the warmer months when water temperature and salinity are optimal for the growth of *V. cholerae*. In regions of the world where it is endemic, cholera typically occurs as a single annual peak in human disease cases. However, bi-annual peaks in cholera cases are typical for the Bengal Delta region^26,50^.

**Figure 3:**
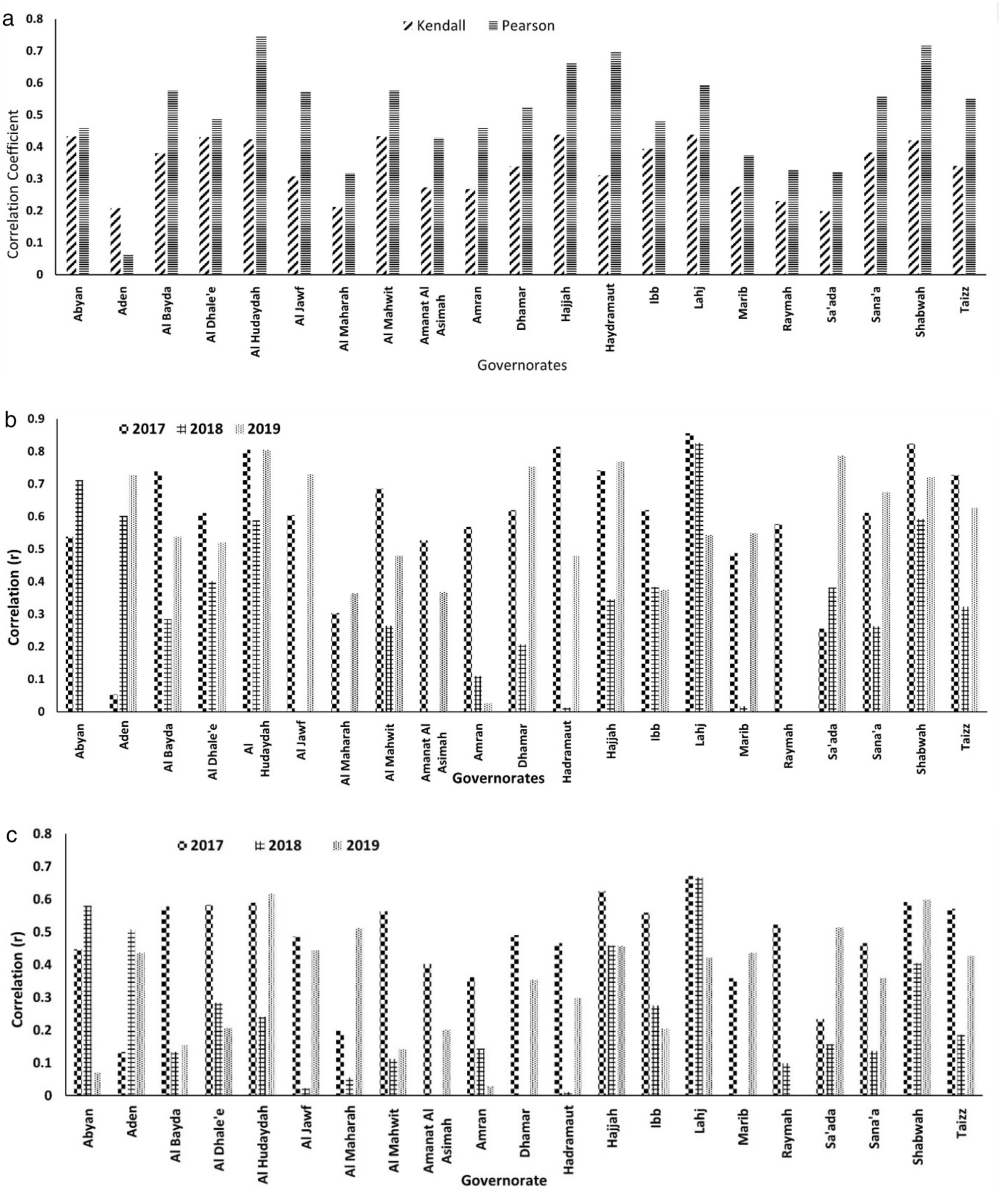
(**a**) Correlation coefficients between cholera cases and risk values for all governorates, all governorates except for Aden exhibited statistically significant (*p* < 0.05) correlation, (**b**) Pearson correlation coefficient between cholera cases and risk values for all governorates for individual years (2017, 2018, 2019) and 19 (of 21), 11 (of 21), and 15 (of 20) governorates are statistically significant in 2017, 2018 and 2019 respectively, (**c**) Kendall Tau correlation coefficient between cholera cases and risk values for all governorates for individual years (2017, 2018, 2019) and 19, 8, and 13 governorates are statistically significant in 2017, 2018 and 2019 respectively.

To evaluate the strength of CRM, a correlation was calculated, at the governorate level, between cholera prevalence from 2017 to 2019 and risk values computed for the same time period, using the parametric (Pearson) and the non-parametric rank correlation coefficient (Kendall Tau scores). The Pearson method showed a significant (*p* < 0.05) positive correlation for all governorates except Aden (Fig. 3a). Similarly, Kendall Tau values were statistically significant (*p* < 0.05) for all governorates, indicating adequate model strength. The three-year correlation analysis provided evidence of overall model performance. However, it may be argued that if effective intervention strategies were employed, such as robust access to WASH, a decline in model performance over those years would have been observed. The CRM trigger module is designed to capture disease initiation in a region. Therefore, unless a new outbreak(s) occurs in a given area, the model performance should decline over time since the transmission dynamics would dictate the spread of cholera in a human population. Accordingly, correlation analysis was performed on a weekly scale for each year. The Pearson correlation for 19 of 21 (2017), 11 of 21 (2018), and 15 of 20 (2019) governorates, respectively, exhibited a significant (*p* < 0.05) association between computed risk and disease prevalence (Fig. 3b). Using Kendall Tau, 19, 8, and 13 governorates showed a statistically significant association for the same years (Fig. 3c). In 2017, the model detected an increased risk for more than 90% of the governorates, with Aden being the only governorate not determined to be at increased risk. Thus, it can be concluded that the results show correlation analysis can be a useful means of determining the strength of the model. During 2018, the model captured *ca*. 45% (combining both Pearson and Kendall Tau), compared to 2019, where *ca*. 68% of the governorates were captured. The decrease in model performance for 2018 is likely an indication of a change in the definition of cholera-like symptoms and/or the intervention strategies employed to mitigate cholera in the region. However, an increase in the model performance was observed for 2019, suggesting new outbreaks in the region compared to the prior year.

### Specificity

Specificity is used here to evaluate the predictive capacity of CRM and is achieved by quantifying causality of model output with cholera prevalence. Causality is quantified using three statistical metrics: accuracy, sensitivity, and specificity, as defined below:

1 $${\text{Accuracy}} = \left( {{\text{t}}_{{\text{p}}} + {\text{t}}_{{\text{n}}} } \right)/\left( {{\text{t}}_{{\text{p}}} + {\text{t}}_{{\text{n}}} + {\text{f}}_{{\text{p}}} + {\text{f}}_{{\text{n}}} } \right)$$

2 $${\text{Sensitivity}} = {\text{t}}_{{\text{p}}} /\left( {{\text{t}}_{{\text{p}}} + {\text{f}}_{{\text{n}}} } \right)$$

3 $${\text{Specificity}} = {\text{t}}_{{\text{n}}} /\left( {{\text{t}}_{{\text{n}}} + {\text{f}}_{{\text{p}}} } \right)$$

**Figure 4:**
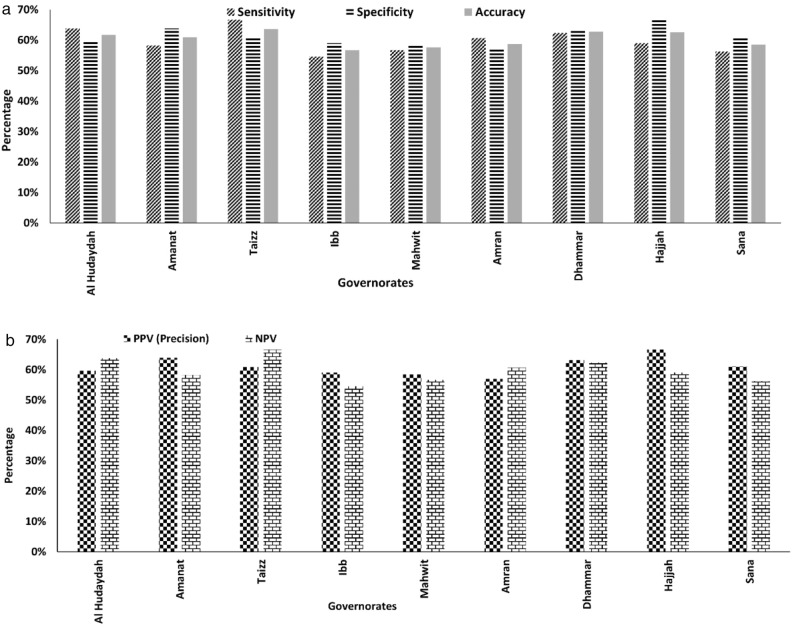
(**a**)Sensitivity [t_p_/(t_p_ + f_n_], Specificity[t_n_/(t_n_ + f_p_], and Accuracy[(t_p_ + t_n_)/(t_p_ + f_p_ + t_n_ + f_n_] of cholera prediction system. (**b**) Positive Predictive Value (PPV) or precision [t_p_/(t_p_ + f_p_)], and Negative Predictive Value (NPV) [t_n_/( t_n_ + f_n_)] of the trigger module of cholera prediction system.

Here an increase in risk is considered a true positive (t_p_) if it captures the increase in reported cases and a decrease in a true negative (t_n_) if it captures the decrease in cases. If the increase in computed risk fails to capture the increase in risk, it is considered a false positive (f_p_); and if a decreased risk fails to capture the decrease in cases, it is considered a false negative (f_n_). The confusion matrix with these variables is provided in Table S1. As shown in Fig. 4a, the cholera risk model met all three criteria for causality more than 60% of the time. Sensitivity and specificity varied from 55 to 67%, with averages of 60% and 61%, respectively, indicating that the model can detect increases and decreases in cholera cases across a given region. Across nine governorates, where more than 100,000 cholera cases had been reported, the model accuracy varied between 57 and 67%, with an average of 60%.

### Biological gradient

Traditionally, the biological gradient is interpreted as a monotonic gradient, indicating direct proportionality of cause of an increase in disease burden with exposure risk. Commensalism of *V. cholerae* with copepods has been established^6,43,51^. *V. cholerae* attaches to the gut and carapace of copepods, with a single copepod capable of harboring up to 10^4^ V*. cholerae* cells^51^. Since cholera is dose-dependent, requiring ingestion of *ca*. 10^3^–10^6^ V*. cholerae* cells to induce severe diarrhea symptomatic of cholera, according to human volunteer studies^52,53^, ingestion of untreated water containing a small number of copepods is sufficient to promote disease. Therefore, if there is contact between human populations and cholera bacteria in the environment, zooplankton blooms can result in numbers of *V*. *cholerae* well above an infectious dose.

It has been argued that BHC should include non-monotonic and more complex relationships between trigger and transmission, with variation in reported cases^46^. Hence, to evaluate the biological gradient in this study, the relationship between change in computed risk, using the model, with change in reported disease prevalence was explored. To evaluate the incidental (biological) gradient of the model, the positive predictive value (PPV; frequently referred to as precision) and negative predictive value (NPV) of the model outcomes were computed. PPV is a fraction of positive computed risk, which can capture positive change in prevalence. This study used an increase in relative risk and reported disease rather than absolute values. NPV is a fraction of negative (decreased) computed risk, which can capture a negative change in prevalence. These two indicators were determined for those governorates for which specificity (causality) of the model had been calculated and were determined as follows:

4 $${\text{Positive predictive value}} = {\text{t}}_{{\text{p}}} /\left( {{\text{t}}_{{\text{p}}} + {\text{f}}_{{\text{p}}} } \right)$$

5 $${\text{Negative predictive value}} = {\text{t}}_{{\text{n}}} /\left( {{\text{t}}_{{\text{n}}} + {\text{f}}_{{\text{n}}} } \right)$$

Using the three years of data, we determined PPV and NPV for the model (Fig. 4b). PPV and NPV values varied between 57 to 67% and 55% to 67%, respectively, with an average of 60%, suggesting that at least 60% of the time, the model correctly responded to an increase or decrease in the number of reported cholera cases. However, for practical use of the cause-effect relationship, temporality is important, with cause preceding effect and lead time.

### Temporality

Epidemiological temporality comprises the duration of exposure and extent of impact in terms of severity or number of incidences. To assess disease risk prediction, lead time is an essential criterion because it provides time to intervene and limit the outbreak's impact. Rainfall has been established as an environmental driver of cholera. In Haiti, increased rainfall was associated with increased cholera risk, with a lag time of up to one month^1,24,35^. Similarly, in Bangladesh, where rainfall/runoff response from upstream catchment areas is about 3–4 weeks, increased rainfall in July has been associated with increased streamflow of major rivers, causing sediment resuspension and attributed to an increase in cholera cases during the month of August^54^. Risk computed in Yemen using the CRM provides a lead time of four weeks from predicted incidence—providing ample time for intervention and mobilization of resources. The hypothesis presented in Fig. 1 shows cholera cases are generally observed four weeks after a period of anomalous warm temperatures, followed by anomalous high precipitation where there is a significant deviation in population behavior, with respect to water use, caused by damaged WASH infrastructure. Importantly, the hypothesis for the temporality of BHC has been supported in several regions in Africa^39^, Asia^40^, and Latin America^22^.

### Consistency

Consistency is an essential parameter of the BHC to ensure the reproducibility of findings across different samples and locations. Our key hypothesis in Fig. 1 argues that damaged WASH infrastructure and a combination of hydroclimatic processes favor conditions for an outbreak of cholera. This cause-and-effect relationship has been observed in many studies^21,24,40^. The hypothesis has been validated for countries in Africa, Asia, and the Americas, reinforcing its reproducibility through its utilization in Yemen. Attributing the cholera outbreak in Peru to El-Niño events in the Central Pacific was one of the earliest precursors to this hypothesis^1^. Studies conducted using data from Bangladesh^16^ and Haiti^22^ report a strong relationship between rainfall and the incidence of cholera. In Bangladesh, cholera occurs annually in a bimodal cycle. The first peak occurs in the spring, and a larger peak occurs following the fall monsoon season. Cholera seasonality also coincides with the warmest temperatures of the year and is reduced to sporadic incidence as the temperature decreases in winter^21^. Haiti has been the main focus of cholera research since the 2010–2011 outbreak, which identified rainfall as a critical driver of the disease in that country^55^. Rainfall can significantly impact the water source, e.g., nutrient concentration, salinity, pH, river level, and freshwater discharge, which affect the growth and persistence of *V*. *cholerae* and its zooplankton host in the environment. Various studies have reported air temperature and precipitation as dominant hydroclimatic variables impacting the occurrence and transmission of cholera in various parts of the world (Table S2).

### Plausibility

Biological plausibility under BHC can be used to assess the association between a putative cause and an observed outcome within the context of existing biological and medical knowledge. In aquatic reservoirs, elevated temperatures can cause a density differential amongst layers of the water column, contributing to the stratification of bacterial populations. In addition, stratification promoted by temperature, dissolved oxygen, pH, and other physical/chemical parameters can determine non-uniform microbial community profiles in the water column contributing to environmental conditions enhancing bacterial growth and multiplication. These conditions are generally favorable for the multiplication of zooplankton, namely copepods, shown by Kaneko and Colwell^6^ to host *Vibrio spp*., including *V. cholerae*. Subsequently, it was shown that employing simple filtration can effectively remove zooplankton and particulate matter, hence attached *V. cholerae,* from drinking water and was used to reduce the number of cholera cases in Bangladeshi villages by more than 50%^43,44^. Collectively, these studies show that copepods serve as host/vector of *V. cholerae*.

Therefore, ecological parameters enhancing the growth and proliferation of cholera bacteria frame the model developed for risk prediction. Heavy rainfall that follows a period of high air temperature aids explosive growth of bacteria in water bodies that serve communities as drinking water source^24^. Thus, an inadequate water supply infrastructure exposes a given population to untreated water. A prime example is Yemen, a Middle Eastern country grappling with war and frequently experiencing floods, with the population lacking proper WASH conditions—considered the dominant sociological cause of the continuing cholera epidemic rampant in most Yemen governorates. Hence, identifying and describing the mechanics of the trigger, a rational clarification of the putative 'black box' between the biology and ecology of an infectious agent and its disease epidemiology, is now possible.

### Coherence

Under BHC, coherence is the idea that experimental findings in the laboratory support epidemiological observations. The hypothesis used in Yemen to predict the cholera risk had exhibited coherence in its application in Yemen and in laboratory findings. The consistency between the epidemiological data and the predicted risk is exhibited through the correlation analysis as shown in Fig. 3. Year 2017 and 2019 exhibited high associations suggesting coherence between the predicted risk and the epidemiological data. While through laboratory studies we found that in the environment, an increase in *V. cholerae* populations was observed in water and plankton samples collected in a longitudinal, multi-year study carried out in the Chesapeake Bay, Maryland, USA. Results showed when the water temperature rose above 19 °C, *V. cholerae* populations in the water column proliferated with the elevated temperatures^56^. Similarly, water samples collected in estuarine zones of the Bengal Delta yielded similar results, confirming enhanced growth of *V. cholerae* in warmer pond water^19^. Huq et al.^36^ showed a 5 °C increase in water temperature resulted in a 30-fold increased risk of a cholera outbreak, with a lag of six weeks. Over the past decades, extreme heat events in Northern Europe have been linked to an increased number of reported *Vibrio* infections^57,58^. Archived samples collected by continuous plankton recorder over 60 years and analyzed using molecular genetic methods by Vezzulli and colleagues^15,59^ showed the global warming trend in sea surface temperatures of the North Sea was strongly associated with proliferation of populations of *Vibrio spp*., including *V. cholerae* and vibriosis in populations inhabiting coastal regions. In fact, among the environmental variables examined, increased sea surface temperature explained ca. 45% of variance in those studies.

Extreme precipitation can also impact *V. cholerae* populations in the environment, with potential to alter salinity profiles and nutrient availability, as well as sea level in coastal waters, with increased freshwater inflow. For example, strong positive correlations between rainfall patterns and cholera epidemics have been observed in Bangladesh^16^, India^20^, Ghana^60^, Cameron^61^, and numerous other locations in Asia, Africa, and South America^62^. Combined with findings from laboratory experiments conducted in different regions of the world (Table 1), these observations comprise a crucial validation based on experimental evidence that supports the hypothesis of this study (Fig. 1).

**Table 1:** Evidence of coherence using BHC.

Authors	Region
Huq et al.^36^	Laboratory environment from the Bay of Bengal
Huq et al.^76^	Laboratory environment from the Bay of Bengal
Skorupski and Taylor ^77^	Laboratory environment
Louis et al.^56^	Laboratory environment from the Chesapeake Bay
Schuhmacher and Klose^78^	Laboratory environment
Singleton et al.^79^	Laboratory environment
Ravel et al.^80^	Laboratory environment
Eiler et al.^81^	Laboratory environment in Baltic Sea
Austin and Swing^82^	Summary of the impact of temperature from various regions
Stauder et al.^83^	Laboratory environment
McCarthy^84^	Laboratory experiments in USA

### Experiment

Various studies have associated hydroclimatic variables with cholera trigger and transmission risk^20,22,26,39^. As discussed above, laboratory-based investigations showed *V. cholerae* thrives in aquatic environments where the water temperature is between 20 and 45°C^63^. Experimental studies have shown an increased risk of cholera when air temperatures are between 19 and 28°C^56,64^, coupled with increasing water entrapment^23,24^. Temperature and precipitation are primary factors in prediction but do not trigger or control the spread of cholera when each is considered independently^65^. The combination of warm temperature converging with heavy rainfall and inadequate WASH infrastructure^45,66^ has a high probability of leading to an outbreak of the disease^24,39^. Furthermore, Colwell and colleagues^1^ demonstrated resuscitation of VBNC *V. cholerae* to the culturable state in the stool, following ingestion of VBNC cells by human volunteers, evidence that non-culturable *V. cholerae* can cause disease. That is, VBNC cells retain virulence potential. Conversion of antigenic *V. cholerae* serotype O1 to non-O1 (as well as non-O1 to O1) has also been demonstrated in laboratory experiments^67,68^, with the conclusion that all *V. cholerae*, regardless of serotype, should be considered potentially pathogenic.

Since *V. cholerae* is a ubiquitous and naturally occurring inhabitant of aquatic environments globally^69,70^, conditions favoring its growth and multiplication suggest that incorporating a single parameter provides, at best, incomplete description of disease trigger and transmission of cholera. The CRM incorporates both temperature and precipitation as hydroclimatic variables, which demonstrated experimentally to have a significant association with the proliferation of the bacterium and cholera.

### Analogy

Analogy of BHC indicates similarities between observations resulting in same delivered outcomes. Unfortunately, cholera outbreaks are a regular phenomenon in regions of the world that are subjected to anomalous precipitation that is also associated with anomalous air temperatures (hydroclimatic conditions)^20,22,24,26^, notably regions with damaged WASH infrastructure^24,40^. Spatial analyses of data from India, Bangladesh, Nepal, Mozambique, Cameroon, Central African Republic, Congo, and Zimbabwe exhibit a similar hydroclimatic pattern related to cholera outbreaks^22,26,27,29,71^. Damaged WASH infrastructure accelerates interaction with *V. cholerae* by increased exposure to lack of safe water, sanitation, and hygiene and the likelihood of waterborne disease. In 2015, implementation of sufficient WASH infrastructure was demonstrated in Nepal to have the potential to reduce the extent of an outbreak, even when hydroclimatic conditions favor an outbreak of cholera^40^. Clearly, WASH infrastructure can be highly effective in controlling the spread of cholera in a population and must be considered in the context of public health, as was the case in Yemen in 2018 (Figure S2).

### Reversibility

As a final criterion of BHC, if the cause is removed, then the effect should disappear as well. Hence, WASH, included in CRM trigger analysis, allows testing reversibility of the model. After the 2015 earthquake, Nepal presented environmental conditions indicative of a massive cholera epidemic^40^. However, the observed outbreak was less than critical and only a few cholera cases were reported. The comparably low number of cholera cases was attributed to the implementation of extremely effective WASH infrastructure and the availability of WASH facilities. The steps that were taken played a crucial role in preventing a massive cholera outbreak in Nepal. The CRM, by incorporating WASH, precipitation, and air temperature, can test for reversibility. Every variable failing to demonstrate positive anomalous variability from the long-term average can be interpreted as indicating minimal cholera risk.

Table 2 summarizes the BHC criteria used to evaluate the CRM trigger module, which provides an assessment of cholera risk at least four weeks in advance (based on previously published results^32^). The motivation for using risk score, rather than prevalence or incidence, is the ability to circumvent missing data during public health emergencies, a situation common during most major cholera outbreaks. Therefore, the risk score remains associated with reported epidemiological data to evaluate the validity of the CRM. In fact, using the model to respond quickly to outbreaks does not eliminate the need to respond to epidemiological data. Instead, it offers a credible risk prediction tool that aims to anticipate when an early response could assist in changing the shape of an epidemiological curve. A critical observation to use outputs from CRM was that there were no protocols available to make decisions for implementing intervention and mitigation efforts by public health officials. It was noted that the majority of humanitarian response to an outbreak of waterborne disease (such as cholera) falls within the reactionary domain implying that the intervention and mitigation activities are generally initiated after an outbreak has been reported in a region. There needs to be a paradigm shift to the anticipatory decision-making process that prediction-based prevention will likely be more effective in reducing the burden of diseases. However, intervention activities in the anticipatory decision-making domain are likely to be significantly different and unique since it may be geared towards preparedness for an impending cholera outbreak (and not for ongoing outbreak), which remains within the scope of future research. Figure 4a and b show results based on environmental conditions of the cholera trigger module related to access to safe water and sanitation. When information on WASH infrastructure becomes available, values for four metrics (Sensitivity, specificity, accuracy, and precision) will improve significantly. Most of the population-centric governorates (Amanat, Amran, Dhamar, Sana'a) were statistically insignificant relative to cholera trigger risk in 2018, most likely an indication that progressive intervention and mitigation were activated and effective.

**Table 2:** Bradford Hill Criteria to evaluate trigger module of the cholera prediction system.

Criteria	Parameter	Fulfillment
Strength	Strong association (correlation) is more causal than weak association	Correlation analysis (Fig. 3)
Consistency	Consistent findings from other studies	Previous studies support the correlation (Huq et al.^24^, Khan et al.^40^, Lipp et al.^21^, Colwell^1^, Hashizume et al.^16^, Khan et al.^22^)
Specificity	Causality of CPS is evaluated through Sensitivity, specificity, and accuracy	Figure 4a
Temporality	Cause occurs before effect	A four-week lead time in hydroclimatic processes was observed to be the cause of cholera
Biological gradient	Higher exposure leads to more public health burden	The gradient analysis was conducted in terms of PPV (precision) and NPV (Fig. 4b)
Plausibility	Mechanism of cause	Previous studies have established precipitation and temperature as the mechanics of survival of cholera bacteria in the environment
Coherence	Epidemiological findings match with laboratory/observational/analytical experiments	Previous studies have determined the presence of cholera bacteria in an aquatic environment (Louis et al.^56^, Neogi et al.^19^)
Experiment	Experimental or analytical evidence	Direct dependence of increase in temperature and precipitation with the increase in cholera risk (Hood et al.^64^, Louis et al.^56^, Huq et al.^24^)
Analogy	Are there any similarities/dissimilarities between the observed association to other processes?	A spatial analysis from India, Bangladesh, Nepal, Mozambique, Cameroon, Central African Republic, Congo, Zimbabwe shows a similar pattern of origin of cholera
Reversibility	Do preventative actions lead to alteration of cause-effect or vice versa?	Preventative actions may have a positive cause-effect impact on the reduction of cholera cases in the year 2018 (Fig. S2)

## Conclusion

This study presents a unique opportunity to reflect upon the future of understanding outbreaks of water-borne diseases where pathogenic growth of *Vibrio spp*. is linked with modalities of water, climate, and environmental processes. Our research results indicate that the solution to water-borne diseases, such as cholera should not be limited within the constraints and bounds of traditional medicinal domains. Rather, a broader approach must be followed with due diligence given to data-driven methodologies aimed at predicting cholera risk in the human population.

We do wish to highlight the importance of using the CRM risk scores rather than the traditional disease model output. The reason is that risk scores are independent of the cholera time series and, therefore, are useful for decision making and developing policies based on the severity of the disease. There are two challenges or limitations of the study: first, the major limitation is inconsistency in the definition of cholera, which was altered in 2018 for Yemen. However, our previous research carried out in Bangladesh^36,54,72^ showed that acute diarrheal disease typically comprises 20 to 30% cholera cases. Second: the availability of global social burden data, including WASH and natural resources. It is important to note that the spatial resolution of the epidemiological data is very coarse, whereas the model output is at 1 km × 1 km. The information available for water and sanitation infrastructure is not efficiently collected; that is, it is not available to be used in our current modeling system.

In summary, the study reported here represents the first to monitor cholera in Yemen with the objective of validating a near real-time cholera prediction model. BHC comprises a series of ten parameters that can be used to provide epidemiological evidence of a causal relationship between public health outcomes and factors influencing the outcome. BHC was effectively employed to assess the performance of the CRM trigger of the cholera prediction system. When epidemiological data on cholera prevalence becomes available on a finer spatial scale, it will be possible to determine cholera hot spots in locations where the human population density is both high and vulnerable to frequent environmental disturbances in water and sanitation. It is concluded that BHC can be used for model evaluation and performance of CRM with accuracy and as shown here, the results of the BHC evaluations are interpreted as being strongly favorable for CRM.

## Methods

We built CRM as an integrated platform, using a heuristic approach, that calculates the risk of the trigger of cholera, model’s detail is provided in the supplementary material. Our previous research created a pathway to the formulation of CRM. The hypothesis was first developed over the Indus River Basin^24^ and defined environmental variables' association with cholera outbreaks. Further, retrospectively, these associations were quantified and validated over Africa^39^, Asia^40^, and Caribbean^22^ regions. Here, utilizing those protocols, we developed CRM, a near real-time cholera risk prediction model, to predict the trigger of a cholera outbreak. Trigger represents those mechanisms stimulating cholera bacteria growth, multiplication, and persistence in the environment, after which under given water insecurity conditions, the interaction of the bacteria with the human population occurs. CRM evaluated cholera in Yemen from 2017 to 2019, as shown in Fig. 2a. CRM comprises a trigger^22,73^ module, which uses data for precipitation, temperature, population, and (WASH) infrastructure to compute a risk score with categorical values of high and low risk of cholera in a given region. The trigger algorithm identifies anomalous temperature and rainfall conditions, providing an assessment of cholera for the following four weeks for a given region. Details of model development and algorithmic architecture have been published elsewhere^40^.

Daily and monthly rainfall data at two different resolutions were obtained from the National Aeronautics and Space Administration (NASA). Monthly rainfall data (0.25° × 0.25°) from the Tropical Rainfall Measuring Mission were employed to compute the long-term average. Daily rainfall data (0.1° × 0.1°) were obtained from the Global Precipitation Mission and used to determine precipitation variation from the long-term average at resampled data points. Daily and monthly data for air temperature on the surface (0.5° × 0.625°) were obtained from the NASA Modern-Era Retrospective analysis Research and Application Version 2 and used to determine temperature variation and deviation from long-term averages. LandScan population data (1 km × 1 km) were obtained from Oak Ridge National Laboratory and used in the model to represent human population (averaged over 24 h) distribution. The CRM outputs were resampled at a spatial resolution of 10 km to provide predicted cholera risk for the following four weeks. Weekly reports of cholera cases at the governorate level, between January 2017 and July 2019, were obtained from the Early Warning, Alert, and Response System and The Assessment Capacities Project^74^. Figure S1 shows total cholera cases across all governorates during 2017, 2018, and the first 28 weeks of 2019.

The trigger model output is a risk score that ranges from high (numerical value of 1) to low (numerical value of 0), inherently different from traditional compartmental disease models where output is usually presented as prevalence or incidence of cholera^22,75^. Pearson (parametric) and Kendall Tau rank (non-parametric) correlation coefficients were used to establish the association between the CRM risk score and cholera cases for all Yemen governorates. For each time point, trigger risk scores were computed and compared with the total number of cholera cases reported during the following four weeks. That is, the efficacy of forecasted cholera risk (four weeks in advance) was evaluated for the trigger module in near real-time. Complementary analyses of sensitivity, specificity, accuracy, precision, and NPV were used to indicate the association between the changes in model risk scores and the change in the number of cholera cases.

## Supplementary Information

Supplementary Information.

## Data Availability

All data generated or analyzed during this study are included in this published article.
