# Supplementary material for: Combating cholera by building predictive capabilities for pathogenic Vibrio cholerae in Yemen
Source: Sci Rep. 2023 Feb 8;13:2255. doi: 10.1038/s41598-022-22946-y (PMC9908932; doi:10.1038/s41598-022-22946-y)
Supplement: Supplementary file 1 — Supplementary Information. [file 41598_2022_22946_MOESM1_ESM.docx]

**Combating cholera by building predictive capabilities for pathogenic *Vibrio cholerae* in Yemen**

***Moiz Usmani, Kyle D. Brumfield, Bailey M. Magers, Juan Chaves-Gonzalez, Helen Ticehurst, Rosa Barciela, Fergus McBean, Rita R. Colwell, Antarpreet Jutla****

**Supplementary Material**

**Model:**

***Trigger Model-*** For our predictive model, we developed an algorithm using precipitation, temperature, and sociological data (as shown in figure 1a) from different sources along with ground information of WASH facilities in Yemen. A pathway was determined using these variables, which states that if a region with damaged WASH facilities, for a specific period, experiences a positive anomalous temperature followed by positive anomalous rainfall with a lag of four weeks, then it will be at high risk of a cholera outbreak. These anomalies for temperature and precipitation were calculated over the long-term average values using equation (a).

$Anomalous Value=Present month value-Long term average value of same month$……..(a)

In this study, the significance of these anomalous variabilities in temperature and precipitation is computed by determining the ratio of these anomalous variations to the standard deviation of respective grid points (standard score). We quantified these anomalous hydroclimatic variables in standard scores to capture the significance of these variabilities and their impact on the prediction of risk maps. The significance of these variables is directly proportional to standard scores; thus, as the scores increases from 1, 2, and 3, the confidence interval increases to 68%, 95% to 99.7%, respectively. Along with the anomalous standard score, the average precipitation of the previous month is used as one of the predictive variables.

**Classification and Weights**- For the trigger model, we used six input variables, namely, average precipitation and positive anomalous variability of precipitation with a lag of one month (four weeks), positive anomalous variability of temperature with a lag of two months (eight weeks), average temperature, population density, and WASH data. These variables are classified on a scale of 0 (minimum) to 4 (maximum), based on the defined microbiologic and hydroclimatic parameters, except for average temperature and sociodemographic instability, which are binary. This classification has been done to mitigate the impact of combining these variables of varying ranges and different numbering systems. In this study, each reclassified input variable is given equal importance, thus was given equal weights. WASH facility in Yemen was considered completely damaged based on information provided by DfID and UNICEF.

**Results:**

Strength

Interestingly, the first report of *V. cholerae* serotype O139 occurred in the chars near the southwestern coastal districts of Bagerhat, Bangladesh, where islands emerge at the end of the monsoon season^1^. Because of poor communication with the mainland, the *V. cholerae* O139 was not reported until December 1992^1^. By September of the following year, the epidemic caused by *V. cholerae* O139 had spread to northern regions of Bangladesh—coinciding with seasonal outbreaks of *V. cholerae* O1^49^.

The bimodal distribution of cholera cases in Bangladesh is annual, with the first peak in the spring and a second, larger peak after the monsoon season in the fall. In Calcutta, India, cases tend to peak between April and June, while in South America, cholera cases occur most frequently during the summer months (January and February)^50^. In Haiti, the number of cholera cases oscillates yearly with an increase during the rainy season (April to October) and a decrease in the dry season^51,52^. Furthermore, with genetic analysis, it has been shown that cholera outbreaks are typically multiclonal^52,53^, suggesting cholera outbreaks typically lack a common source and the causative agent has a broad distribution.

When environmental conditions are unfavorable, *V. cholerae* is able to enter a protective, (VBNC) state whereby the bacterium becomes metabolically dormant, enhancing survivability and persistence in the environment^13,54^. Subsequently, when environmental conditions are again favorable for growth and multiplication, typically triggered by temperature, salinity, and nutrient conditions, VBNC cells regain cultivability and virulence^13,35^. Copepods feed on phytoplankton, and a high correlation between phytoplankton blooms and an increased number of copepods with cholera cases has been observed^14^. The increased abundance of phytoplankton followed by zooplankton blooms also promotes nutrient-rich waters favorable for the growth of *V. cholerae*^49,55^. Clearly, the limits of endemicity are consistent and suggest a biogeographical pattern of endemic disease promoted by interaction of the environment and *V. cholerae* ecology. Hence, remote sensing of phytoplankton blooms, coupled with temperature and precipitation, can be used as an effective predictor of cholera epidemics^25,56,57^.

Temporality:

Moreover, de Magny and colleagues^21,56^ have proposed a lag of up to 4 weeks from the occurrence of increased zooplankton abundance and subsequently increased presence of *V. cholerae*. Hence, temperature, salinity, rainfall, and plankton have proved important in *V*. *cholerae* ecology. Our previous studies quantified environmental, climatic, and sociological processes influencing cholera outbreak in a population^25^.

**Figures**

| 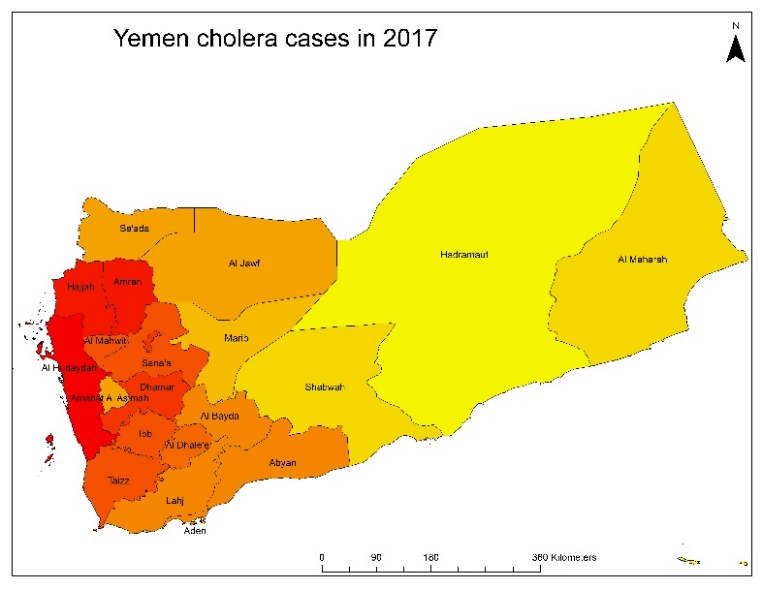 |
| --- |
| 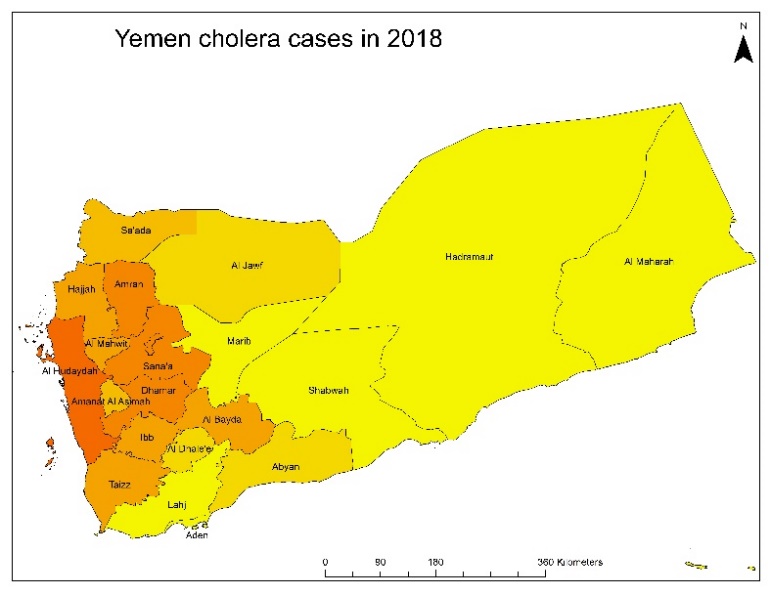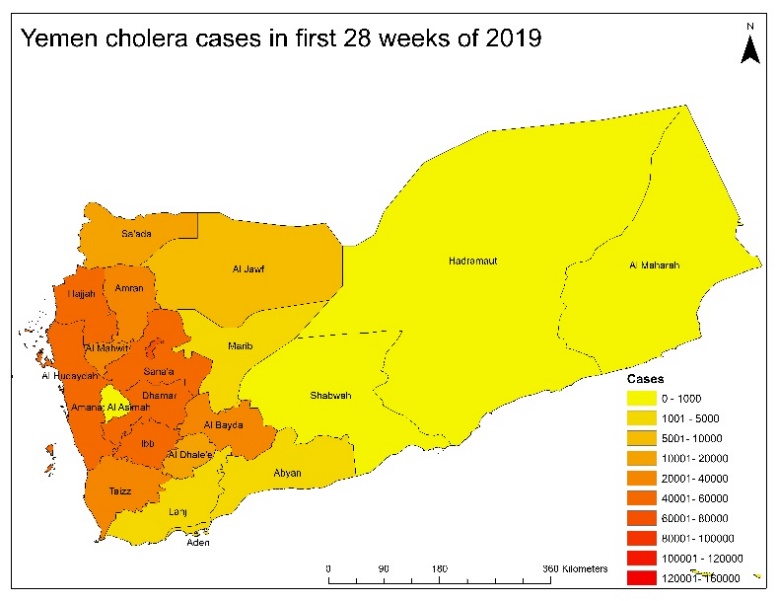 |
| **Figure S1:** Total cholera cases in Yemen in 2017, 2018 and first 28 weeks of 2019 (maps are generated using ESRI’s ArcMap version 10.7: https://desktop.arcgis.com/en/arcmap/10.7/get-started/setup/arcgis-desktop-quick-start-guide.htm).  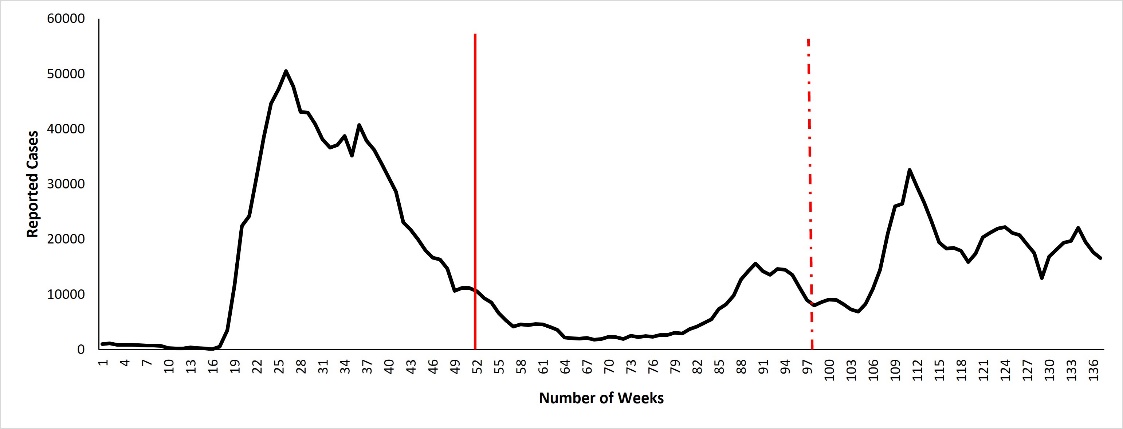  **Figure S2**: Total number of cholera cases from 2017 to 2019 in Yemen, the first vertical (solid) line and second vertical (dotted) line marks the end of year 2017 and 2018 respectively. |

**Tables**

**Table S1**: Confusion matrix for various governorates in Yemen. Here, Fn is false negative, Fp is false positive, Tn is true negative, and Tp is true positive.

| Conditions | Al Hudaydah | Amanat | Taizz | Ibb | Mahwit | Amran | Dhammar | Hajjah | Sana |
| --- | --- | --- | --- | --- | --- | --- | --- | --- | --- |
| F_n_ | 21 | 33 | 21 | 30 | 29 | 24 | 26 | 25 | 28 |
| F_p_ | 25 | 26 | 27 | 25 | 27 | 28 | 25 | 18 | 23 |
| T_n_ | 37 | 46 | 42 | 36 | 38 | 37 | 43 | 36 | 36 |
| T_p_ | 37 | 46 | 42 | 36 | 38 | 37 | 43 | 36 | 36 |

**Table S2**: Evidence of consistency using BHC

| Authors | Region | Citation score | Climatic variable |
| --- | --- | --- | --- |
| Pascual et al 2000 | Bangladesh | 587 | Temperature |
| Lobitz et al 1999 | Bangladesh | 516 | Temperature |
| Rose et al 2001 | Bangladesh and Peru | 492 | Temperature |
| Huq et al 2005 | Bangladesh | 342 | Temperature, Rainfall |
| Griffith et al 2006 | Africa | 252 | Rainfall |
| Louis et al 2003 | USA | 214 | Rainfall, Temperature |
| Rinaldo et al 2012 | Haiti | 150 | Rainfall |
